# Supplementary material for: Fast-Dissolving Nifedipine and Atorvastatin Calcium Electrospun Nanofibers as a Potential Buccal Delivery System
Source: Pharmaceutics. 2022 Feb 4;14(2):358. doi: 10.3390/pharmaceutics14020358 (PMC8874982; doi:10.3390/pharmaceutics14020358)
Supplement: Supplementary file 1 [file pharmaceutics-14-00358-s001.zip › pharmaceutics-1562712-supplementary.pdf]

pharmaceutics

Supplementary Materials: Fast-Dissolving Nifedipine and Atorvastatin Calcium Electrospun Nanofibers as a Potential Buccal Delivery System

Hassa A. Alshaya, Ahmed J. Alfahad, Fatemah M. Alsulaihem, Alhassan H. Aodah, Abdullah A. Alshehri, Fahad A. Almughem, Haya A. Alfassam, Ahmad M. Aldossary, Abdulrahman A. Halwani, Haitham A. Bukhary, Moutaz Y. Badr, Salam Massadeh, Manal Alaamery and Essam A. Tawfik

Nifedipine and Atorvastatin Calcium Determination and Quantification using High-Performance Liquid Chromatography (HPLC).

In order to quantify nifedipine and atorvastatin calcium in the drug-loaded core-shell nanofibers, an HPLC method was developed, in which the calibration curve of the separation of both drugs is shown in Figure 6. Both the regression equation and R^2 were determined to be y=10.796x − 0.2228 (R^2 = 0.9999) for nifedipine and y=41.047x − 11.784 (R^2 = 0.9999) for atorvastatin calcium, indicating the successful separation of both drugs and the excellent linearity of this method.

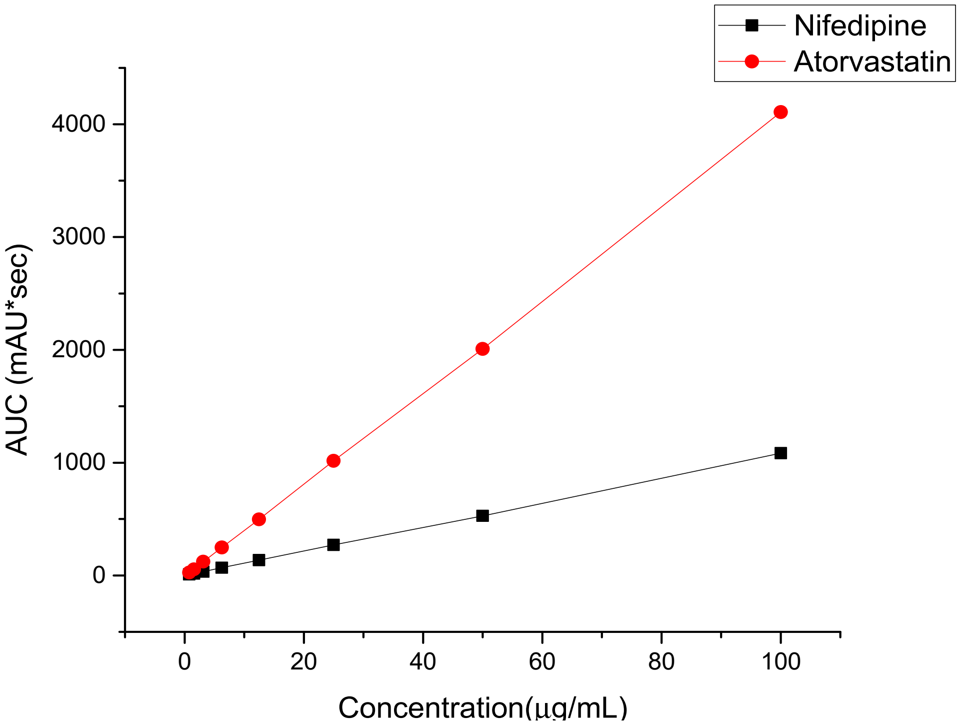

Figure S1. The HPLC calibration curves of nifedipine and atorvastatin calcium, showing excellent linearity ($R^2 \geq 0.9999$) for both drugs. The regression equation of nifidepine is y=10.796x − 0.2228; while it is y=41.047x − 11.784 for atorvastatin calcium.
